# Evolutionary Musicology Meets Embodied Cognition: Biocultural Coevolution and the Enactive Origins of Human Musicality

**DOI:** 10.3389/fnins.2017.00519

**Published:** 2017-09-29

**Authors:** Dylan van der Schyff, Andrea Schiavio

**Affiliations:** ^1^Faculty of Education, Simon Fraser University, Burnaby, BC, Canada; ^2^Faculty of Music, University of Oxford, Oxford, United Kingdom; ^3^Institute for Music Education, University of Music and Performing Arts, Graz, Austria; ^4^Department of Music, The University of Sheffield, Sheffield, United Kingdom; ^5^Centre for Systematic Musicology, University of Graz, Graz, Austria

**Keywords:** origins of music, biocultural coevolution, music cognition, enactive cognition, dynamical systems theory

## Abstract

Despite evolutionary musicology's interdisciplinary nature, and the diverse methods it employs, the field has nevertheless tended to divide into two main positions. Some argue that music should be understood as a naturally selected adaptation, while others claim that music is a product of culture with little or no relevance for the survival of the species. We review these arguments, suggesting that while interesting and well-reasoned positions have been offered on both sides of the debate, the nature-or-culture (or adaptation vs. non-adaptation) assumptions that have traditionally driven the discussion have resulted in a problematic *either/or* dichotomy. We then consider an alternative “biocultural” proposal that appears to offer a way forward. As we discuss, this approach draws on a range of research in theoretical biology, archeology, neuroscience, embodied and ecological cognition, and dynamical systems theory (DST), positing a more integrated model that sees biological and cultural dimensions as aspects of the same evolving system. Following this, we outline the enactive approach to cognition, discussing the ways it aligns with the biocultural perspective. Put simply, the enactive approach posits a deep continuity between mind and life, where cognitive processes are explored in terms of how self-organizing living systems enact relationships with the environment that are relevant to their survival and well-being. It highlights the embodied and ecologically situated nature of living agents, as well as the active role they play in their own developmental processes. Importantly, the enactive approach sees cognitive and evolutionary processes as driven by a range of interacting factors, including the socio-cultural forms of activity that characterize the lives of more complex creatures such as ourselves. We offer some suggestions for how this approach might enhance and extend the biocultural model. To conclude we briefly consider the implications of this approach for practical areas such as music education.

## Introduction

The debate over the origins and meaning of music for the human animal is one of the most fascinating areas of inquiry across the sciences and humanities. Despite the diversity of perspectives on offer, however, this field has traditionally been guided by approaches that see adaptation by natural selection as the central mechanism driving evolutionary processes (Huron, 2001; for a discussion see Tomlinson, 2015). This extends to the brain, which is often understood as a computing machine that evolved to solve the kinds of problems faced by our prehistoric ancestors in their everyday lives (see Anderson, 2014). Importantly, this “adaptationist” orientation posits a rather strict separation between the products of natural selection (i.e., adaptations) and those of culture. Because of this, evolutionary musicologists have often been faced with something of a dichotomy: Music tends to be seen *either* as a naturally selected adaptation that has contributed directly to our survival as a species, *or* as a product of culture with little or no direct connection to our biological heritage (see van der Schyff, 2013a; Tomlinson, 2015; Killin, 2016a, 2017). Various arguments have emerged in support of each position (more on this below; see Pinker, 1997; Huron, 2001; Mithen, 2005; Patel, 2008; Honing et al., 2015). Moreover, the influence of the computational model of mind has tended to focus research and theory in music cognition toward a complex information-processing hierarchy limited to the brain (Sloboda, 1985; Deutsch, 1999; Huron, 2006; Levitin, 2006). This is sometimes discussed in terms of discrete cognitive modules that have been naturally selected to perform specific tasks related to the survival of the species (Fodor, 1983; Pinker, 1997; Coltheart, 1999), leading some scholars to postulate 1:1 mappings between anatomical brain regions and musical functions (Peretz and Coltheart, 2003; cf. Altenmüller, 2001). While this research has indeed produced a number of important insights, it has arguably tended to downplay the role of the environmentally situated body in the development of musicality as a cognitive domain (see Clarke, 2005; Johnson, 2007).

In recent years, new perspectives have emerged that place more focus on the embodied, ecological, and dynamical dimensions of musical cognition (e.g., Borgo, 2005; Clarke, 2005, 2012; Reybrouck, 2005, 2013; Leman, 2007; Jones, 2009; Krueger, 2013; Maes et al., 2014; Moran, 2014; Laroche and Kaddouch, 2015; Godøy et al., 2016; Schiavio and van der Schyff, 2016; Schiavio et al., 2016; Lesaffre et al., 2017). Recent research has also tended to weaken the modular hypothesis by emphasizing the plastic and self-organizing properties of the (musical) brain (Jäncke et al., 2001; Pantev et al., 2001; Münte et al., 2002; Lappe et al., 2008; Large et al., 2016). The past two decades have also seen the development of a “biocultural” hypothesis for the origins and nature of the musical mind that looks beyond the traditional nature-culture dichotomy (Cross, 1999, 2003; Killin, 2013, 2016a,b, 2017; van der Schyff, 2013a,b; Tomlinson, 2015). This approach draws on a range of research in theoretical biology, neuroscience, embodied and ecological cognition, and dynamical systems theory (DST), positing a more integrated model that sees biological and cultural dimensions as aspects of the same evolving system. Here the origin of music is not understood within a strict adaptationist framework. Rather, it is explained as an emergent phenomenon involving cycles of (embodied) interactivity with the social and material environment.

Our aim in the present article is to contribute to the theoretical discussion supporting the biocultural hypothesis by considering it through the lenses of the enactive approach to cognition. This perspective first emerged in the work of Varela et al. (1991) and has been developed more recently across a range of contexts (Thompson, 2007; Stewart et al., 2010; Colombetti, 2014; Di Paolo et al., 2017). Most centrally, the enactive approach posits a deep continuity between mind and life, where cognitive processes are explored in terms of how self-organizing living systems enact relationships with the environment that are relevant to their survival and well-being. It highlights the embodied and ecologically situated nature of living agents, as well as the active role they play in their own developmental processes. Importantly, the enactive approach sees cognitive and evolutionary processes as driven by a range of interacting factors, including the socio-cultural forms of activity that characterize the lives of more complex creatures such as ourselves (Malafouris, 2008, 2013, 2015). We suggest, therefore, that it may help to extend the biocultural hypothesis in various ways.

We begin by providing a brief overview of some key positions in the field of evolutionary musicology, discussing how many tend to adhere to the “nature-or-culture” dichotomy mentioned above. We then outline the biocultural hypothesis, reviewing supporting research and theory in theoretical biology, neuroscience, and ecological and embodied cognition. Here we place a special focus on Tomlinson's (2015) approach as, for us, it represents the current state of the art in the field. While we are largely in agreement with his position, we suggest that future work could benefit from exploring a wider range of perspectives in embodied-ecological cognition. With this in mind, we then discuss the enactive approach and consider how it might enhance the biocultural perspective. More specifically, we suggest that the enactive view could offer theoretical support and refinement to Tomlinson's claim that the origins of the musical mind should be sought for in the embodied dynamics of coordinated action that occurred within the developing socio-material environments of our ancestors—and not first in terms of cognitive processes involving (quasi-linguistic) representational mental content. Following this, we consider how the recently emerged 4E approach—which sees cognition as *embodied, embedded, enactive*, and *extended—*aligns with the biocultural perspective, offering some tentative possibilities for how this framework might guide future research associated with the biocultural approach. To conclude we briefly consider the implications this perspective may have for thought and action in practical musical contexts (e.g., music education). Before we begin, we would also like to note that although the enactive approach is being explored across several disciplines (see Stewart et al., 2010), it has only recently been adopted in musical contexts (Borgo, 2005; Silverman, 2012; Krueger, 2013, 2014; Matyja and Schiavio, 2013; Elliott and Silverman, 2015; Loaiza, 2016; Schiavio et al., 2016). Therefore, this article may also contribute to the development of the enactive perspective for musical research and theory more generally.

## Evolutionary musicology and the dichotomy of adaptation

An important point of discussion in evolutionary musicology concerns whether musicality can be considered as a bona fide adaptation, or if it is better understood as a product of culture (Huron, 2001; Davies, 2012; van der Schyff, 2013a; Lawson, 2014; Honing et al., 2015; Killin, 2016a, 2017). Some researchers (including Darwin, 1871) have drawn on comparisons with music-like behavior in other animals, suggesting an adaptive function for music in mate selection and territorial display in our prehistoric ancestors (see Miller, 2000). It has been argued, however, that although music-like behavior in non-human animals (e.g., bird song) may well be a product of natural selection, these traits are not *homologous* with human music making, but rather are *analogous* (Pinker, 1997; Hauser and McDermott, 2003). Because of this, it is claimed that comparative studies involving more phylogenetically distant species may not have great relevance for understanding the biological origins of human musicality (McDermott and Hauser, 2005; but see Fitch, 2006). Additionally, evidence of “musical” behaviors in our closest primate relatives is often understood to be sparse. For some scholars, this suggests there was no properly musical phenotype prior to modern humans in the hominin line (Huron, 2001; Justus and Hutsler, 2005; Patel, 2008).

Such arguments have been used to support claims that music should not be conceived of as an adaptation, but rather as a product of culture (e.g., Sperber, 1996; Pinker, 1997). Here it is posited that music is dependent on cognitive structures (e.g., modules) and abilities that evolved to support properly adaptive functions in our ancestors (e.g., language, auditory scene analysis, habitat selection, emotion, and motor control—for a discussion see Trainor, 2015). Perhaps the strongest version of this approach is found in Pinker (1997), who argues that music is an “invention” designed to “tickle” these naturally selected aspects of our cognitive and biological nature. Music itself, however, has no adaptive meaning: From an evolutionary point of view, it is the auditory equivalent of “cheesecake”—a cultural invention that is pleasurable, but biologically useless. In line with this, it is suggested that music might be a kind of exaptation—where the original (i.e., adapted) function of a trait becomes co-opted to serve other purposes^1^ (Davies, 2012). Thus, as Sperber (1996) posits, music may be understood as “parasitic on a cognitive module the proper domain of which pre-existed music and had nothing to do with it” (p. 142).

By contrast, other researchers have suggested the existence of cognitive modules that appear to be specialized for musical functions. For example, Peretz (1993, 2006, 2012) research in acquired *amusia* has led her to (cautiously) posit an innate music-specific module for pitch processing, suggesting that music may be as “natural” as language (Peretz, 2006). Such claims are countered by Patel (2008), who argues that evidence indicating the existence of adapted music specific modules may in fact be explained by (ontogenetic) developmental processes, whereby cortical areas become specialized for certain functions through experience (e.g., via processes of “progressive modularization”; see Karmiloff-Smith, 1992). However, while Patel (2008, 2010) maintains that musicality in humans is not a “direct target” of natural selection, he also acknowledges the profound biological and social benefits associated with musical activity, claiming that music is a powerful “transformative technology of the mind” (Patel, 2008, p. 400–401). Here Patel discusses how musical experience may lead to long-lasting changes in brain structure and processing (e.g., though neuroendocrine effects). Interestingly, he also notes that the phenomenon of infant babbling, the anatomy of the human vocal tract, and the fixation of the FOXP2 gene, might be indicative of adaptations that originally supported both language and vocal music (Patel, 2008, p. 371–372). However, he suggests that because language appears to emerge more quickly and uniformly in humans, and because the lack of musical ability does not appear to entail significant biological costs, these factors are better understood to support the adaptive status of language. In brief, he posits that musical processing is a “by-product” of cognitive mechanisms selected for language and other forms of complex vocal learning (see also Patel, 2006, 2010, 2012).

These last claims are questioned by those who argue that they may reflect a rather narrow perspective on what musicality entails—e.g., the assumption that musical activity necessarily requires special forms of training, or that music is a pleasure product to be consumed at concerts or through recordings (for discussions see Small, 1999; Cross, 2003, 2010; van der Schyff, 2013a,b; Honing et al., 2015). With regard to this point, ethnomusicological and sociological research has revealed musical activity around the world to be central for human well-being—it is inextricable from work, play, social life, religion, ritual, politics, healing, and more (Blacking, 1973, 1995; Nettl, 1983, 2000; DeNora, 2000). Moreover, in many cultural environments music is highly improvisational in character, and the acquisition of musical skills begins in infancy and develops rapidly, often without the need for formal instruction (Blacking, 1973; Cross, 2003; Solis and Nettl, 2009). It has also been suggested that because certain physical and cognitive deficits need not hinder survival and well-being in modern Western society, certain “musical” impairments may go almost completely unnoticed (van der Schyff, 2013a). Likewise, music's relevance for human survival across evolutionary time has been considered in terms of its importance for bonding between infants and primary caregivers, and between members of social groups (Benzon, 2001; Tolbert, 2001; Dissanayake, 2010; Dunbar, 2012). Musical developmental processes appear to begin very early on in life (Parncutt, 2006) and researchers have demonstrated the universal and seemingly intuitive way caregivers create musical (or music-like) environments for infants through prosodic speech and lullabies (Dissanayake, 2000; Trehub, 2003; Falk, 2004). Along these lines, Trevarthen (2002) has proposed that humans possess an in-born “communicative musicality” that serves the necessity for embodied inter-subjectivity in highly social beings such as ourselves (see also Malloch and Trevarthen, 2010).

In all, it is argued that the wide range of activities associated with the word “music” may have immediate and far-reaching implications for survival and socialization for many peoples of the world, as it may have had for our prehistoric ancestors (see Blacking, 1973; Mithen, 2005). And indeed, the archeological record shows evidence of musical activity (i.e., bone flutes) dating back at least 40,000 years (Higham et al., 2012; Morley, 2013). Such concerns drive the “musilanguage” theory put forward by Mithen (2005) and others (Brown, 2000; Lawson, 2014), where both music and language are understood to have developed from a “proto-musical ancestor” that evolved due to selective pressures favoring more complex forms of social behavior—e.g., enhanced types of communication associated with foraging and hunting, mate competition, increased periods of child rearing (soothing at a distance), and more complex forms of coordinated group activity (Dunbar, 1996, 2003, 2012; Cross, 1999, 2003; Falk, 2000, 2004; Balter, 2004; Bannan, 2012). Here it is also suggested that musical behavior may have contributed to the development of shared intentionality and Theory of Mind (ToM) in modern humans, which in turn permitted the rapid development of cultural evolution and the emergence of modern human cognition (Tomasello, 1999; Tomasello et al., 2005).

## The biocultural hypothesis

Thus far, we have offered only a brief outline of some of the main positions in the discussion over the status of music in human evolution. We would like to suggest, however, that although many important and well-reasoned accounts have emerged on both sides of the debate, the nature-or-culture perspective that appears to frame this discussion renders both sides somewhat problematic. On one hand, arguing that music is primarily a product of culture may tend to downplay its deep significance for human well-being, as well as the rather rapid and intuitive ways it develops in many cultural contexts. Indeed, as we have just considered, these manifold developmental and social factors are taken to be indicative of the biological relevance of music for the human animal. On the other hand, arguments for music as an adaptation (e.g., Mithen, 2005; Lawson, 2014) often tend to posit a singular adaptive status for what is in fact a complex phenomenon that spans a wide range of biological, social, and cultural dimensions (Tomlinson, 2015).

In line with such concerns, other scholars (Cross, 1999, 2001, 2003; Killin, 2013, 2016a; van der Schyff, 2013a,b; Currie and Killin, 2016) have offered alternative “biocultural” approaches to the nature and origins of human musicality—where the question of whether *either* biology *or* culture should account for deeply social and universal human activities that require complex cognitive functions (e.g., music) is replaced by a perspective that integrates the two. For example, Cross (1999) suggests that musicality is an emergent activity—or “cognitive capacity”—that arises from a more fundamental human proclivity to search for relevance and meaning in our interactions with the world. It is claimed that because of its “multiple potential meanings” and “floating intentionality” music provides a means by which social activity may be explored in a “risk free” environment, affording the development of competencies between different domains of embodied experience and the (co)creation of meaning and culture (Cross, 1999, 2003). Tomlinson (2015) develops similar insights, arguing that what we now refer to as “language” and “music” began with more basic forms of coordinated socio-cultural activity that incrementally developed into more sophisticated patterns of thought, activity, and communication (see also Morley, 2013). Moreover, such activities are understood to have transformed environmental niches over time (Sterelny, 2014; Killin, 2016a, 2017) and with them the behavioral possibilities (affordances) of the hominines who inhabited them through recursive cycles of feedback and feedforward effects.

In all, this orientation suggests a way through the traditional nature-or-culture dichotomy discussed above. In doing so, however, it necessarily draws on models of evolution and cognition that differ from those that have traditionally guided evolutionary musicology. In line with this, Tomlinson's (2015) approach develops Neo-Peircean perspectives in semiotics (e.g., Deacon, 1997, 2010, 2012), exploring how embodied and indexical forms of communication may in fact underpin our linguistic and musical abilities both in evolutionary and ontogenetic terms. As we discuss below, this is further supported by work in theoretical biology associated with developmental systems theory, studies of musical and social entrainment (rhythm and mimesis), and insights from ecological psychology and embodied cognition.

### Looking beyond adaptation

Tomlinson (2015) argues that although music-as-adaptation perspectives all reveal important aspects of why music is meaningful for the human animal, they are also problematic when they tend to assume a “unilateral explanation for a manifold phenomenon” (p. 33; see also Killin, 2016a). That is, because music takes on so many forms, involves such a wide range of behavior, and serves so many functions, it seems difficult to specify a single selective environment for it. And thus, these traits sit “uneasily side by side, their interrelation left unspecified” (p. 33). To be clear, this does not in any way negate the claims regarding the social and developmental meanings of music. These biologically relevant traits do exist, but they are just too numerous and complex to be properly described in terms of an adaptation (at least not in the orthodox sense of the term). Because of this, Tomlinson (2015) claims that we must be careful about how we frame evolutionary questions—and especially those regarding complex behaviors such as music and language—lest we fall into the reductive theorizing associated with “adaptationist fundamentalism.” He thus argues that dwelling on the question of the adaptive status of music has had the effect of “focusing our sights too narrowly on the question of natural selection alone—and usually a threadbare theorizing of it, at that” (p. 34).

With this in mind, the developmental systems approach to biological evolution posits a useful alternative perspective (see Oyama et al., 2001). In contrast to the one-directional schema that characterizes more traditional frameworks (where evolution is understood to involve *adaptation to* a given environment), developmental systems theory presents a more recursive and relational view, where organism and environment are understood as mutually influencing aspects of the same integrated system. Here evolutionary processes do not entail the adaptation of a species' phenotype to a fixed terrain, but rather “a dynamic interaction where other species and the non-living environment take part” (Tomlinson, 2015, p. 35). In other words, this approach explores the complex ways genes, organisms, and environmental factors—including behavior and (socio-cultural) experience—interact with each other in guiding the formation of phenotypes and the construction of environmental niches (Moore, 2003; Jablonka and Lamb, 2005; Richerson and Boyd, 2005; Malafouris, 2008, 2013, 2015; Laland et al., 2010; Sterelny, 2014). As such, it eschews the classic nature-nurture dichotomy, preferring instead to examine the interaction between organism and environment as a recursive or “dialectical” phenomenon (Lewontin et al., 1984; Pigliucci, 2001), where no single unit or mechanism is sufficient to explain all processes involved.

**Figure 1:**
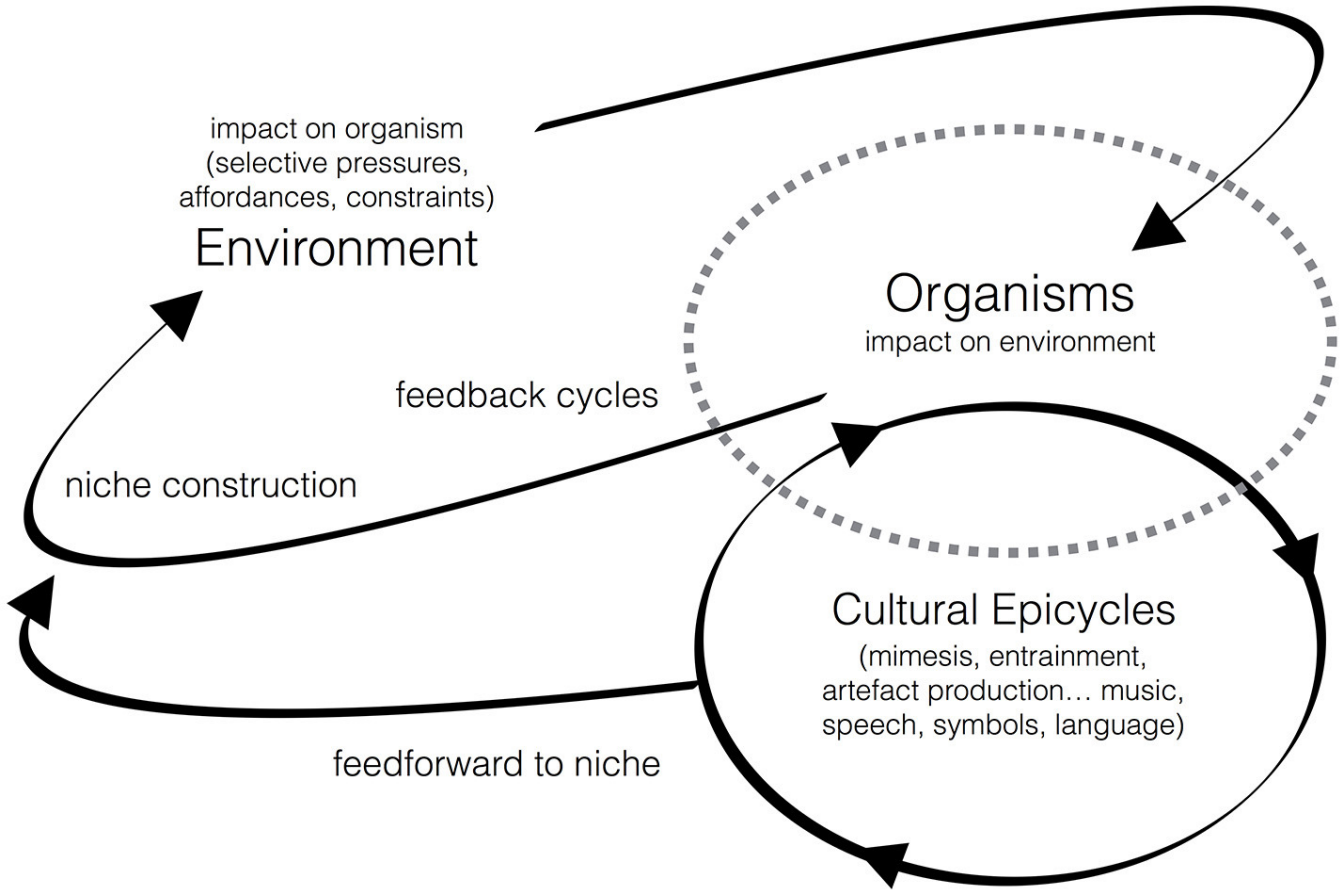
Describes the cyclical process of biocultural coevolution (adapted with permission from Tomlinson, 2015, p. 46–47). Note that this depicts the most general level of description and does not show the more micro-level “cycles within cycles” that occur, for example, within the intra-organism milieu. These include the patterns of muscular, emotional-affective, neural, and metabolic activity that influence the expression of genes and gene groups over various timescales. This, in turn, helps to guide developmental processes and behavior that impacts the environmental niche.

Importantly, the organism is understood here to play an active role in shaping the environment it *coevolves with*—its activities feedback into and alter the selective pressures of the environmental niche. This, in turn, affects the development of the organism, resulting in a co-evolutionary cycle that proceeds in an ongoing way. Socio-cultural developments add additional epicycles involving patterns of behavior that can sometimes hold stable over long periods of time (see Figure 1). These are passed on inter- and intra-generationally through embodied mimetic processes (more on this below; see also Sterelny, 2012). While such epicycles necessarily emerge from the coevolution cycle, they may, once established, develop into self-sustaining patterns of behavior that develop relatively independently. However, the effects of these cultural epicycles may feedforward into the broader coevolutionary system resulting in additional alterations to environmental conditions and shifts in biological configurations (e.g., gene expression and morphological changes—see Wrangham, 2009; Laland et al., 2010; Skinner et al., 2015; Killin, 2016a).

The making and use of tools is offered as a primary example of what such cultural epicycles might entail (Tomlinson, 2015). The archeological record contains many examples of bi-face stone hand axes that were made by our Paleolithic ancestors. These tools are remarkably consistent in their functional and aesthetic qualities, implying method and planning in their manufacture (Wynn, 1996, 2002). However, it is now thought that the production of these axes entailed a “bottom up” process based on the morphology and motor-possibilities of the body, unplanned emotional-mimetic social interaction, and the affordances of the environment (Gamble, 1999; Davidson, 2002). In other words, it is argued that the emergence of Paleolithic technologies did not involve abstract or representational forms of thought (e.g., a mental template, or “top down” thinking)—a capacity these early toolmakers did not possess (but see Killin, 2016b, 2017). Nor were they the result of genetically determined developmental programs. Rather, they are thought to have originated, developed, and stabilized primarily through the dynamic interaction between living systems and the material environments they inhabited and shaped (Ingold, 1999). It is suggested that such self-organizing forms of social-technological behavior provided the grounding from which more complex cultural activities like music emerged much later (Tomlinson, 2015). To better understand how this could be so, we now consider the mimetic nature of these pre-human social environments, and how this may give clues to the origins of music in coordinated rhythmic behavior.

### Mimesis, entrainment, and the origins of music in rhythm

In social animals, attention tends to be turned “outwards” toward the world and the activities of others (McGrath and Kelly, 1986). This entails the capacity to observe, understand, and emulate the actions of conspecifics. It is suggested that in our Paleolithic ancestors these mimetic processes allowed increasingly complex chains of actions to be passed on from one individual or generation to the next (Leroi-Gourhan, 1964/1993; Gamble, 1999; Ingold, 1999). This involved the enactment of culturally embedded “action loops” (see Donald, 2001; Tomlinson, 2015) that depended on a basic proclivity for forms of social *entrainment*.

The phenomenon of entrainment may be observed in many ways and over various timescales in both biological and non-biological contexts (de Landa, 1992; Clayton et al., 2005; Becker, 2011; Knight et al., 2017). Most fundamentally, it is understood in terms of the tendency for oscillating systems to synchronize with each other^2^. Accordingly, biological and social systems can be conceived of as dynamically interconnected systems of oscillating components (from metabolic cycles to life cycles, from single neuron firing to regional patterns of activity in the brain, from individual organisms to social groups and the broader biological and cognitive ecology; McGrath and Kelly, 1986; Oyama et al., 2001; Varela et al., 2001; Ward, 2003; Chemero, 2009). Importantly, the components of such systems influence each other in a non-linear or recursive way. As such, organism and environment are not separate domains, but rather aspects of “one non-decomposable system” that evolves over time (Chemero, 2009, p. 26). Moreover, the development of coupled systems is guided by local and global constraints that allow the system to maintain stability—to be resistant to perturbations, or to regain stability once a perturbation has occurred. This is, of course, crucial for living systems, which must maintain metabolic functioning within certain parameters if they are to survive.

Such self-organizing processes result in “emergent properties”—relationships, structures, and patterns of behavior that may remain consistent over long temporal periods, or that may be subject to transformation due to shifts in local and global constraints of the system. The mathematical techniques associated with DST have aided researchers in modeling such phenomena. Here patterns of convergence (stability) in the state of the system are contrasted with areas exhibiting entropy (instability; de Landa, 1992). This is often represented as a topographic “phase-space” that describes the possible states of a given system over time—periods of convergence in the trajectories of the system are represented as “basins of attraction” (Abraham and Shaw, 1985; Chemero, 2009). A “phase transition” occurs when new patterns of convergence arise (i.e., new attractor layouts). Researchers associated with developmental systems theory (above) use DST methods to model the evolutionary trajectories of coupled organism-environment systems, mapping dynamic patterns of stability and change as functions of constraint parameters (see Oyama et al., 2001).

DST is also used to examine how social animals bring their actions in line with those of other agents—and with other exogenous factors—by “dynamically attending” to the environment through sight, sound, movement, and touch (McGrath and Kelly, 1986; Large and Jones, 1999). This results in the enactment of coordinated forms of behavior that can occur both voluntarily and involuntarily. Emotional-affective aspects may also come into play here. For example, when a stable pattern is disrupted, entropy emerges in the system and a negative affect may result. The (living) system then self-organizes toward regaining stability, resulting in a positive effect. It is suggested that the action loops associated with Paleolithic toolmaking emerged from these forms of social entrainment—where dynamic couplings between various trajectories in the social environment led to increasingly stable patterns of behavior (basins of attraction) in the cultural epicycle. This permitted the mimetic transmission of cultural knowledge without the need for symbols, referentiality, or representation (see Tomlinson, 2015, p. 75).

Interestingly, the idea of dynamic attending has been explored empirically in the context of musical (i.e., metrical, rhythmic) entrainment (Large and Jones, 1999; Jones, 2009; Large et al., 2015). Tomlinson (2015) suggests that such dynamical models may help to reveal the distant origins of musical rhythm in the mimetic, emotional, and sonic-social environments jointly enacted by the coordinated (entrained) motor patterns of early toolmakers. This insight is supported by a range of current research into the evolution of rhythmic behavior (Fitch, 2012; Merchant and Bartolo, 2017; Ravignani et al., 2017). Indeed, evolutionary musicology has often tended to explore the origins of music in terms of its vocal dimensions (i.e., music as pitch/song production and its relationship to spoken language), and has thus had to wrestle with the issues associated with complex vocal learning, and its apparent absence in other primates. The focus on rhythm, however, has shown similarities between animal and human behavior (Fitch, 2010; Patel and Iversen, 2014; Merchant et al., 2015; Bannan, 2016; Iversen, 2016; Wilson and Cook, 2016). A large number of papers have also explored the deep relationship between rhythmic behavior and social cohesion in both human and non-human subjects (e.g., Large and Gray, 2015; Yu and Tomonaga, 2015; Tunçgenç and Cohen, 2016; Knight et al., 2017). Additionally, recent studies by Ravignani et al. (2016a) have modeled the cultural evolution of rhythm in the lab. This research shows how, when presented with random percussive sounds, participants tend to develop structured and recurrent rhythms from such information, and that these patterns continue to develop through subsequent generations of participants who are asked to imitate the rhythms of previous generations. Interestingly, the rhythmic patterns that emerged in this study display six statistical universals found across different musical cultures and traditions. This aligns with the conception of cultural transmission based on mimesis and entrainment just discussed. It also implies that the enactment of musical (or music-like) behavior may not be traceable solely to the genome, but rather arises due to a more general propensity to structure acoustical experience in certain ways (see also Fitch, 2017).

Here it should be noted that the biocultural approach also develops a theory about the origins of vocal musicality, albeit one that is deeply connected to the rhythmic factors just described. This entails the development of a repertoire of “gesture-calls” similar to those found in modern primates and many mammalian species (grunts, pants-hoots, growls, howls, barking, and so on; see Tomlinson, 2015, p. 89–123). These do not involve the abstract, symbolic-representational, and combinatorial properties employed by modern languages. Rather, they are tightly coupled with the same mimetic, emotional, and embodied forms of communication that characterize pre-human tool-making. It is suggested that the vocal expressions associated with these gesture-calls reflected the sonic aspects (rhythmic and timbral) of these environments, the motor-patterns of production, as well as the gestural and social rhythms (e.g., turn taking, social entrainment) that developed within the cultural ecology. In line with this, studies show connections between rhythmic capacities and the development of vocal forms of communication, including language (Cummins and Port, 1996; Cummins, 2015; Bekius et al., 2016; Ravignani et al., 2016b). As an aside, it is also posited that the process of knapping may have resulted in specific forms of listening (Morley, 2013, p. 120), and that the resonant and sometimes tonal qualities of stones and flakes may have afforded music-like play with sound (Zubrow et al., 2001; Killin, 2016a,b)^3^. In brief, these rhythmic forms of behavior may have led to proto-musical and proto-linguistic forms of communication that arose simultaneously.

However, as Tomlinson (2015) notes, “half a million years ago there was no language or musicking” (p. 127). While many music-relevant anatomical features were in place by this period, there is no evidence that these hominins possessed the more complex forms of combinatorial thinking required for the hierarchical structuring of rhythm, timbre, and pitch associated with musical activity (i.e., the kind of thinking that is also needed to build tools specifically intended for musical use, such as bone flutes). Rather, it is posited that proto-musical and proto-linguistic communications were initially limited to deictic co-present interactions (in-the-moment face-to-face encounters that integrated gesture and a limited number of vocal utterances) that incrementally developed into more complex sequences of communicative behavior. Over time, this led to the enactment of increasingly sophisticated forms of joint action and social understanding (Dunbar, 1996, 2003; Knoblich and Sebanz, 2008; Sterelny, 2012). Such developments in the cultural loop fed forward into the coevolutionary cycle, allowing the environmental niche to be explored in new ways, affording previously unrecognized modes of engagement with it. This, in turn, altered selective pressures, leading to incremental phase transitions in the dynamics of the system, where previous constraints were weakened and new behavioral-cognitive phenotypes became possible. By the Upper Paleolithic period, the growing influence of the cultural epicycle favored an enhanced capacity to understand the actions and intentions of others and the related capacity to think “offline,” “top down,” or “at a distance” from immediate events (Bickerton, 1990, 2002; Carruthers and Smith, 1996; Tomasello, 1999). These developments allowed for the marshaling of material and social resources in new ways, leading to the creation of more complex artifacts (e.g., musical instruments), as well as more sophisticated types of cultural activity (e.g., ritual) and communication, including the hierarchical and combinatorial forms required for language and music as we know them today^4^.

### Plastic brains

The biocultural approach sees (musical) cognition as an emergent property of situated embodied activity within a developing socio-material environment. Because of this, it requires a rather different view of cognition than the information-processing model associated with an adapted (modular) brain (e.g., Fodor, 1983, 2001; Tooby and Cosmides, 1989, 1992; Pinker, 1997; Barrett and Kurzban, 2006). Indeed, if evolutionary processes do not involve adaption to a pre-given environment, but rather require the active participation of organisms in shaping the environments they *coevolve with*—where “selection” and “adaptation” are now understood in a contingent and dynamically cyclical context—then it seems reasonable to suggest that cognitive processes might not depend on genetically programmed responses or be reducible to a collection of fixed information-processing mechanisms in the brain. Rather, they might entail more plastic and perhaps non-representational characteristics that reflect the dynamic integration of brains, bodies, objects, and socio-cultural environments (for similar arguments see Malafouris, 2008, 2013, 2015).

In line with such concerns, scholars are questioning whether the notion of modularity continues to have much relevance for understanding the complexities of the human brain (e.g., Uttal, 2001; Doidge, 2007; Anderson, 2014). For example, it is suggested that brain regions that appear to consistently correlate with specific processes, such as Broca's area and syntax, represent vast areas of the cortex that may in fact develop multiple overlapping or interlacing networks, the manifold functions of which may appear evermore fine-grained and plastic as neural imaging technology becomes more refined (Hagoort, 2005; Poldrack, 2006; Tettamanti and Weniger, 2006; Grahn, 2012). In relation to this, recent research suggests the existence of “global systems” that function in a flexible and context-dependent manner (see Besson and Schön, 2012, p. 289–290). These do not work independently of any other information available to the brain and are thus non-modular (i.e., they are not discrete). Additionally, research into various levels of biological organization is showing that biological and cognitive processes develop in interaction with the environment—e.g., that epigenetic factors play a central role in the expression of genes, and that the formation of neural connections unfolds as a function of context (Sur and Leamey, 2001; Uttal, 2001; Van Orden et al., 2001; Lickliter and Honeycutt, 2003; Panksepp, 2009). In short, the idea that brain and behavior are best understood as linear systems decomposable into discrete modules and corresponding functions is being replaced by more plastic^5^ and dynamically interactive perspectives. Such insights have contributed to the growing view that music cognition is the result of *non-modular* cognitive developmental processes that are driven by a more general attraction to coordinated forms of social behavior (Trehub, 2000; Trehub and Nakata, 2001-2002; Trehub and Hannon, 2006; see also Drake et al., 2000).

Because of this, recent decades have seen researchers turn to “connectionist” models to account for essential cognitive functions such as (musical) perception and learning (see Desain and Honing, 1991, 2003; Griffith and Todd, 1999; Clarke, 2005). Likewise, Tomlinson discusses the connectionist approach as a way of understanding how the embodied-ecological processes of mimesis and social entrainment contributed to the development of music and language. Put simply, the connectionist strategy does not rely on the idea of fixed modules, but rather on the fact that when simple devices (such as individual neurons) are massively interconnected in a distributed way such connections may change and grow through “experience”—when neurons tend to become active together, their connections are reinforced and vice versa (Hebb, 1949). Such connectivity is thought to result in the emergence of complex sub-systems of activity as well as global convergences that produce system wide properties. This is often modeled using DST and can also be understood in terms of the oscillatory dynamics mentioned above (see Chemero, 2009).

### Embodied minds

While the connectionist approach was initially seen as an alternative to the computational orientation, more recent modeling has revealed the ability of complex connectionist networks to simulate syntactic, representational, and combinatorial cognitive processes (see Smolensky, 1990; Bechtel, 2008)—i.e., those required by the “adapted brain” hypothesis. Such developments are attractive for some researchers as they allow for the assumed computational-representational nature of cognition to remain while accommodating the growing evidence around brain plasticity and dynamism (Chalmers, 1990; Smolensky, 1990; Dennett, 1991; Clark, 1997; on compositionality see van Gelder, 1990). However, others maintain that because the brain's connectivity cannot be separated from its dynamic history of coupling with the body and the environment, living cognition is not best understood as strictly limited to in-the-brain computations and representational content (Varela et al., 1991; Thompson, 2007; Chemero, 2009; Hutto and Myin, 2012).

To better understand what this means for the biocultural approach to music's origins, it may be useful to consider Tomlinson's (2015, p. 129–139) reading of Cheney and Seyfarth's (2008) research into the social lives of baboons. As Tomlinson notes, observations of baboon vocal and gestural interactions lead Cheney and Seyfarth to suggest that the social behavior of these animals is indicative of an underlying hierarchical and syntactic-representational cognitive structure—one that is continuous with the Fodorian notion of “the language of thought” or “mentalese” (a process of non- or pre-conscious symbolic manipulation in the brain according to syntactic rules). This, they suggest, may reveal a deep evolutionary connection between linguistic processing and social intelligence—where linguistic-computational processes are thought to underpin social cognition even if no spoken or symbolic language is present (as with baboons and our pre-human ancestors; cf. Barrett, forthcoming). However, Cheney and Seyfarth also hint at another possibility, where a more plastic and dynamic connectionist framework comes into play. The idea here is that once a system learns to organize itself in various ways, the patterns it develops can be recognized by the system in association with various things and relationships and thus may be said to “represent” them^6^. For this reason, connectionist processes are sometimes thought to be “sub-symbolic” in that they provide a link between biological processes at lower levels and representational processes at higher ones (Varela et al., 1991, p. 100; Smolensky, 1988). In line with this, Cheney and Seyfarth (2008) suggest that as animals engage with their environments neural networks could be reinforced leading to multimodal forms of “distributed neural representation” (p. 241; see also Barsalou, 2005; Tomlinson, 2015, p. 133). As Tomlinson (2015) points out, this implies something less abstract and more concretely embodied and ecological:

[A] *quite literal re-representing, a solidifying, affirming, salience-forming set of neural tautologies*. There is no reliance on abstracted social identities such as those humans conceive, on a mysterious language of mind that does the representing, or on baboon comprehension of causality, proposition, and predication. In their place are the accretion of intrabrain and interbrain networks and the responses they enable in face of situations that are both familiar and less so. Networks are, within sheer biological constraints, products of environmental affordances, forged through the repeated patterns of an organism's interaction with the socio material surroundings. […] All the intricacy Cheney and Seyfarth find in baboon sociality may well be explained […] without recourse to anything like mentalese (p. 135–136; italics original).

Similarly, when Tomlinson (2015) refers to the mimetic nature of the developing proto-musical environments, he clarifies that the action loops associated with this may indeed be representational, but not in the sense of mental templates or propositions. Rather, following Donald (2001), Tomlinson comments that the notion of “representation” employed here may entail little more “than the rise to salience of an aspect of a hominin's environment—in this case an enacted sequence of physical gestures imprinting itself in neural networks that fire again when repeated. Or […] a set of interconnected neural oscillations” (p. 73–74).

It is suggested that this revised conception of representation might be more conducive to understanding cognition across a wider range of developmental and phylogenetic contexts. The problem with applying the more traditional approach associated with computational psychology is that it tends to encourage a kind of “reverse engineering, retrospectively projecting human capacities onto earlier hominins or onto nonhuman species understood as proxies for our ancestors” (Tomlinson, 2015, p. 138). This critique resonates with the work of Barrett (2011), who discusses our tendency to construct highly anthropomorphic views of other life forms and how this can lead to false understandings—not only of their cognitive capacities, but also of the nature and origins of human minds. Similarly, it is argued that the traditional assumption that “cognition” necessarily involves some form of linguistic competence (syntax, propositional thought, symbolic representation, and other forms of abstract “mental gymnastics”) has tended to overshadow the more fundamental embodied and emotional aspects of living meaning making in human cognition (Johnson, 2007). This extends to music, which over the past three decades has been examined with a special emphasis on its relationship to linguistic capacities in cognitive and evolutionary contexts (Patel, 2008; Rebuschat et al., 2012; van der Schyff, 2015).

Now, all of this is not meant to imply that research into the (cognitive and evolutionary) relationship between music and language should be abandoned. This is an important area of inquiry and should continue to be investigated. However, other developmental and socio-cultural factors are receiving growing attention from researchers. This includes accounts that explore the dynamic, ecological, and embodied nature of musical experience (e.g., Large and Jones, 1999; Reybrouck, 2005; Leman, 2007; Krueger, 2013; van der Schyff, 2015; Godøy et al., 2016). As we began to consider above, while music and language both involve hierarchical and combinatorial forms of thought, it may be that both emerge from more domain general capacities and proclivities related to the ways embodied-affective relationships are generated within socio-material environments (Johnson, 2007). For some scholars, this implies that the symbolic-representational and propositional forms of cognition associated with language may be derivative rather than primary (see Hutto and Myin, 2012, 2017). As such, the origins of cognition might not be found in brain-bound computations and symbolic representations, but rather in the self-organizing dynamics associated with biological development itself—in the cycles of action and perception that are directly linked to an organism's ongoing history of embodied engagement with its environment. This recalls the coevolution cycle discussed above, but it may also be considered in the context of ontogenesis—e.g., how infants enact meaningful realities through embodied and affective interactivity with their socio-material niche (see Bateson, 1975; Service, 1984; Dissanayake, 2000; Reddy et al., 2013).

Such insights are not lost on Tomlinson (2015), who highlights the continuity between the embodied activities of Paleolithic tool makers and cognition as such—where cognition might in fact be rooted in interactions with the environment that over time result in increasingly complex extensions of individual embodied minds into the broader cognitive ecology (e.g., via mimesis and social “rhythmic” entrainment). Here Tomlinson also entertains the possibility that the self-organizing (or “self-initiating” as he sometimes refers to it) nature of the activities discussed above might not need to be understood in representational terms at all. However, he does not go much further than this general suggestion. This is perhaps somewhat surprising as he does, here and there, draw on the notion of “affordances” and the field of ecological psychology it is associated with—an explicitly non-representational approach to cognition in its original version (Gibson, 1966, 1979; more on this shortly).

Once Tomlinson outlines the deeply embodied, ecological, and socially interactive precursors of musical behavior, he then turns to explain music cognition using generative (e.g., Lerdahl and Jackendoff, 1983) and prediction- or anticipation-based models (e.g., Huron, 2006) that focus on the (internal) processing of musical stimuli and the behavioral responses they lead to. These approaches are relevant to the discussion as they focus on the more abstract and combinatorial ways the modern human mind processes musical events. We would like to suggest, however, that future contributions might benefit by exploring a wider range of perspectives drawn from embodied cognitive science and related perspectives in music cognition. With this in mind, we now turn to discuss how insights associated with the enactive approach to cognition might help to support and advance many of the claims made by Tomlinson (2015) and the biocultural approach more generally.

## The enactive perspective

The enactive approach to cognition was originally introduced by Varela et al. (1991) as a counter to the then dominant information-processing model of mind and the adaptationist approach to biological evolution^7^. Like the biocultural model, it develops the insights of developmental systems theory and DST, and is inspired by the work of Gibson (1966, 1979). Gibson's “ecological psychology” asks us to rethink the relationship between cognitive systems and their environment. As Chemero (2009) discusses, this can be understood in terms of three main tenets. The first posits that perception is direct (i.e., it is not mediated by representational mental content). The second argues that perception is not first and foremost for information gathering, but is for the guidance of action—for actively engaging with the world. Following from these, the third tenet claims that perception is of “affordances”—or the possibilities for action offered by the environment in relation to the corporeal complexity of the perceiving organism (e.g., a chair affords sitting for a child or an adult, but not for an infant or a fish; Gibson, 1979).

While sympathetic with the three core tenets of the Gibsonian approach, some scholars suggest that the conception of affordances associated with it is problematic when it implies that they are intrinsic features of the environment (e.g., Varela et al., 1991, p. 192–219; for a discussion see Chemero, 2009, p. 135–162). This, it is argued, does not give enough attention to the active role living creatures play in shaping the worlds they inhabit, leading “to a research strategy in which one attempts to build an ecological theory of perception entirely from the side of the environment. Such a research strategy ignores not only the structural unity of the animal but also the codetermination of animal and environment” (Varela et al., 1991, p. 204–205). In brief, the enactivist perspective posits a revised interpretation of affordances that more clearly integrates corporeal dimensions and the engaged perceptual activity of cognitive agents (Varela et al., 1991; see also Nöe, 2006; Chemero, 2009; Barrett, 2011; Schiavio, 2016). As we discuss next, this approach allows for a view of cognition that is not wholly driven by the environment—nor by internal representations—but rather by the embodied activity of living agents. As such, it may allow us to further develop the corporeal and ecological concerns that drive the biocultural model.

### Where there is life there is mind

One of the most central claims of the enactive perspective concerns the deep continuity between mind and life, where cognition is understood to originate in the self-organizing activity of living biological systems (Maturana and Varela, 1980, 1984; Varela et al., 1991; Thompson, 2007; Di Paolo et al., 2017). Most primarily, this involves the development and maintenance of a bounded metabolism (Jonas, 1966; Bourgine and Stewart, 2004; Thompson, 2007), but it also requires the (meta-metabolic) ability of the organism to move and interact with the environment in ways that are relevant to its survival (van Duijn et al., 2006; Egbert et al., 2010; Di Paolo et al., 2017; Barrett, forthcoming). Furthermore, because such fundamental life-processes occur under precarious conditions (Kyselo, 2014), they cannot be fully understood in an indifferent way. Rather, basic cognitive activity is characterized by a “primordial affectivity” that motivates relevant action (Colombetti, 2014). By this view, a living creature “makes sense” of the world through affectively motivated action-as-perception and, in the process, constructs a viable niche (Weber and Varela, 2002; Di Paolo, 2005; Reybrouck, 2005, 2013; Colombetti, 2010; Di Paolo et al., 2017). This involves the *enactment* of affordances—which are conceived of as emergent properties associated with the dynamic (evolutionary and ontogenetic) history of structural coupling between organisms and their environments^8^ (Varela, 1988; Varela et al., 1991; Chemero, 2009; Barrett, 2011; Schiavio, 2016). Importantly, such basic sense-making processes do not involve the representational recovery of an external reality in the head (i.e., mental content). Rather, they are rooted in direct embodied engagement with the environment (Varela et al., 1991; Thompson, 2007)^9^.

In brief, the enactive approach explores cognition in terms of the self-organizing and adaptive sense-making activities by which organisms enact survival-relevant relationships and possibilities for action (i.e., affordances) within a contingent milieu (Thompson, 2007). This constitutes the fundamental cognitive behavior of living embodied minds. Moreover, this perspective traces a continuity between the basic affectively motivated sense-making of simpler organisms and the richer manifestations of mind found in more complex biological forms (Di Paolo et al., 2017). In other words, where the meaningful actions of single-celled and other simple creatures are associated with factors related to nutrition and reproduction, more complicated creatures will engage in ever richer forms of sense making activity and thus exhibit a wider range of cognitive-emotional behaviors (Froese and Di Paolo, 2011). For social animals, this may include “participatory” forms of sense-making that involve the enactment of emotional-affective and empathic modes of communication between agents and social groups (mimesis), and that coincide with the development of shared repertoires of coordinated action (entrainment; see De Jaegher and Di Paolo, 2007; Di Paolo, 2009). With this in mind, we suggest that an enactive framework may provide a useful way of understanding human musical activities as continuous with, but not reducible to, the fundamental forms of self-organizing and emotionally driven action-as-perception that characterize living (participatory) sense-making more generally (van der Schyff, 2015; Loaiza, 2016; Schiavio and De Jaegher, 2017)^10^. As such, it appears to be well positioned to support and extend the biocultural model.

### Enactivism meets the biocultural perspective

The enactive approach to cognition aligns with the biocultural model in several ways. Both draw on developmental systems theory and DST. And both embrace a circular and co-emergent view of organism and environment, as well as a deeply embodied perspective on cognition. Because the enactive approach traces cognition to the fundamental biological concerns shared by all forms of life, it may also help us avoid the anthropomorphizing tendencies noted above (e.g., imposing language-like capacities on non- or pre-human animals; but see De Jesus, 2015, 2016; Cummins and De Jesus, 2016), and thus better understand how cognitive capacities rooted in bodily action might ground the development of music and other cultural activities (Barrett, 2011; Tomlinson, 2015).

In connection with this, researchers drawing on enactivist theory are using DST models to examine bio-cognitive processes in terms of the non-linear couplings that occur between:

- the *body*—the development of muscular linkages and repertoires of corporeal articulation.
- the *brain*—the emergence of patterned or recurrent (i.e., convergent) trajectories in neural activity.
- the *environment*—the enactment of stable relationships and coordinated behavior within the socio-material ecology.

This approach is being explored across a range of areas (see Fogel and Thelen, 1987; Laible and Thompson, 2000; Hsu and Fogel, 2003; Camras and Witherington, 2005), including, for example, emotion research (Lewis and Granic, 2000; Colombetti, 2014), studies of social cognition and inter subjectivity (for a detailed discussion see Froese, forthcoming), and musical creativity (Walton et al., 2014, 2015). We suggest that similar approaches might be employed in conjunction with existing knowledge of early hominin anatomical and social structure, evidence from the archeological record, as well as comparative studies with other species and existing musical activities. This could also be developed alongside recent studies of how musical environments and behavior affect the expression of genes and gene groups, and how this might recursively influence behavioral and ecological factors (see Bittman et al., 2005, 2013; Schneck and Berger, 2006; Laland et al., 2010; Kanduri et al., 2015; Skinner et al., 2015).

Additionally, while recent theory associated with “radical enactivism” (Hutto and Myin, 2012) argues that so-called “basic minds” do not themselves possess any form of representational content, it also suggests that culture and language impose certain constraints that result in cognitive activities that may be understood as content bearing (this echoes the suggestion introduced above regarding the possible non-primary or “secondary” status of representational cognition; see Hutto and Myin, 2017). The explanatory advantages of this approach are currently a subject of debate. Nevertheless, the insights that arise from this discussion might shed new light on the cultural epicycles discussed above. As Tomlinson (2015) points out, although musical activity is not fundamentally symbolic or representational itself, it necessarily occurs and develops within cultural worlds of symbols and language. Put simply, the debate surrounding radical enactivism could offer new perspectives on how, over various developmental periods, cultural being might simultaneously constrain, and be driven by, the non-symbolic, social-affective, and embodied forms of cognition that characterize musical activity.

Another important possibility for how the enactive orientation might contribute to the biocultural approach involves the recently developed 4E framework, which sees cognition in terms of four overlapping dimensions—*embodied, embedded, enactive*, and *extended* (Menary, 2010a; Newen et al., 2017). The *embodied* dimension explores the central role the body plays in driving cognitive processes. This is captured, for example, in the description of the early Paleolithic tool making societies, where the reciprocal influences of sight, sound, and coordinated movement lead to the production of artifacts with specific characteristics. Such forms of embodied activity also formed the basis from which more complex forms of thought and communication emerged later. As we also considered, the biocultural model explores how such embodied factors arise in specific environments, leading to stable and recurrent patterns of activity where bodily, neural, and ecological trajectories converge. This highlights the *embedded* dimension, which concerns the ecological and socio-cultural factors that co-constitute situated cognitive activity. The biocultural model explores this in terms of the sonic, visual, tactile and emotional-mimetic nature of the niches enacted by our early ancestors, as well as the growing influence of the cultural epicycle on the cognitive ecology. The *enactive* dimension, as we have seen, concerns the self-organizing nature of living systems, and describes the active role organisms play in shaping the environments they inhabit. Such modes of activity (which are described as “sense-making”) are explored over a range of timescales (brief encounters, ontogenesis, evolutionary development), closely aligning with the coevolutionary feedback cycle discussed above. As enactivists equate “sense-making” with “cognition” (Thompson, 2007; De Jaegher, 2013), it may be argued that mental life cannot be limited to the brains or bodies of organisms: It extends into the environments in which cognitive processes play out. In line with this, the *extended* dimension explores how many cognitive processes involve coupling with other agents (mimesis, social entrainment, participatory sense-making) or with non-biological objects or cultural artifacts (tools, notebooks, musical instruments; see Menary, 2010b; Malafouris, 2013, 2015). While Tomlinson (2015) makes no mention of enactivism or this 4E framework, he does, as we have seen, discuss how cognitive processes emerged and developed in our Paleolithic ancestors through embodied activity that was situated within a milieu that they actively shaped. He also argues that such activity necessarily involved the coordination of multiple agents and the “extension” of individual minds into the socio-material environment. We suggest, therefore, that a 4E approach might be useful in terms of organizing theoretical concepts and for framing and interpreting relevant empirical research.

The 4E framework is currently being developed by a handful of scholars in association with musical cognition (e.g., Krueger, 2014, 2016; Schiavio and Altenmüller, 2015; van der Schyff, 2017; Linson and Clarke, forthcoming). It is also explored in biological contexts by Barrett (2011, 2015a,b, forthcoming) as an alternative to the brain bound (and arguably anthropomorphizing) approach of traditional computationalism. Additionally, the 4E approach aligns with, and could be used to integrate, the corporeal, neural, and environmental levels of investigation associated with contemporary DST research in musical contexts. Therefore, it could help model how these factors contributed to the development of musical behavior in pre- and early human societies. Likewise, this approach might also have interesting implications for the laboratory modeling of cultural rhythmic transmission. As we began to discuss above, experiments by Ravignani et al. (2016a) examine how individuals trying to imitate random drumming sequences learn from each other in independent transmission chains—where the attempts of one participant become the training set for the next subject. This research aligns with the biocultural and enactive perspectives when it suggests that cultural development is not the product of genetic programming, but is guided by more general dynamical processes and constraints that allow for a range of possibilities. A 4E approach might develop the parameters of such studies to include the manipulation of social environmental (i.e., embedded + extended) factors—possibly exploring how groups of participants (rather than chains of individual drummers) collaboratively make sense of their sonic environments and develop rhythmic patterns in real time, and how the shared environments that result are transmitted and developed (enacted) by the following cohort. Additionally, it might be interesting to introduce different instruments and methods of sound making it to the environment to see how this affects the results. Lastly, a 4E approach could also include the analysis of video and audio recordings to better understand the relationship between the (embodied) motor, sonic, and socio-material factors involved in the enactment of “rhythmic cultures”^11^. If it is indeed the case that it is joint bodily action that drove cognitive and cultural processes in our ancestors, then it would be interesting to see how drumming movements shape shared learning environments, and how they develop into new more structured ones (more efficient and easier to imitate) as the rhythmic patterns are transmitted.

## Conclusion

We have offered here only a few tentative possibilities for how the enactive and 4E orientation might extend the biocultural approach to the origins and nature of human musicality. We hope that the ideas we have discussed here will inspire future work that explores this relationship more fully. Along these lines, readers may be interested to consider recent work by Malafouris (2008, 2013, 2015), who develops enactive and 4E principles to better understand how brains, bodies, and objects interact to form cognitive ecologies. Malafouris expands the idea of neural plasticity discussed above to include the domain of objects, tools, and culture. In doing so he posits a notion of “metaplasticity” that demands an “historical ontology” of different forms of material engagement (Malafouris, 2013, 2015). This is considered at the intersection of neuroscience, archeology, 4E cognition, and approaches to biological evolution that are closely aligned with developmental systems theory. In many ways, Malafouris' perspective sums up the interests and aspirations of the biocultural approach. He writes,

I propose to accept the fact that human cognitive and emotional states literally comprise elements in their surrounding material environment. Our attention, therefore, should shift from the distinction of “mind” and “matter” or “in” and “out,” toward developing common relational ways of thinking about the complex interactions among brain, body, and world. If we succeed, traditional ways of doing cognitive science should change, and the change will stretch far beyond the context of cognitive archaeology and human evolution (Malafouris, 2015, p. 366).

With this in mind, we would like to close by briefly mentioning some ontological and ethical implications an enactive-biocultural model might have for practical areas like music education. If music is neither a pleasure technology, nor the result of some strict adaptationist process—but rather a biocultural phenomenon rooted in the dynamics of joint action—then the ways we approach it in practice (e.g., music education, musicology, performance, music therapy, and so on) should reflect this fundamental existential reality. In other words, this approach opens a perspective on what it means to be and become musical that is no longer based in prescriptive developmental processes, adapted cognitive modules, and correspondence to pre-given stimuli (e.g., music as the reproduction of a score; see Small, 1999). Instead, it highlights the plastic, creative, situated, participatory, improvisational, embodied, empathic, and world-making nature of human musicality. It may therefore offer support to a growing number of theorists who argue that we have tended to rely on disembodied, depersonalized, and highly “technicist” approaches to musical learning (Regelski, 2002, 2016; Borgo, 2007; Elliott and Silverman, 2015), and that this orientation has reduced the ontological status of music students, teachers, listeners, and performers to mere responders, consumers, and reproducers (van der Schyff et al., 2016). Although this cannot be explored in detail here, it is an example of how alternative perspectives on the evolution and nature of human (musical) cognition could inspire new ways of thinking in practical areas. In all, then, we hope that the biocultural and enactive approaches will continue to be developed in musical contexts to gain richer understandings of the origins and meaning of musicality for the human animal.

## Author contributions

DvdS developed the main body of text. AS provided suggestions and comments that were implemented in the final version.

### Conflict of interest statement

The authors declare that the research was conducted in the absence of any commercial or financial relationships that could be construed as a potential conflict of interest.

## Footnotes

^1^The term “exaptation” refers to changes in the function of a given physiological or behavioral trait in the process of the biological evolution of an organism. The classic example is bird feathers, which originally evolved for thermoregulation, but were later co-opted for mating-territorial display, catching insects, and then flight. The developmental systems approach discussed below complicates the causal relation of adaptations and exaptations. Here they stand not in a linear sequence, but rather in a cyclical relationship, where the new uses of an adaptation associated with the exaptation may lead to secondary adaptations and so on (see Gould and Vrba, 1982; Anderson, 2007). Referring to the relationship between adaptations and exaptations Tomlinson (2015) writes, “the first are not necessarily prior to the second, since behaviors originating as exaptations might alter selective pressures in ways leading to new adaptations” (p. 36).

^2^A simple example of this is found in the way wall mounted pendulums mutually constrain one another, resulting in synchronization or “entrainment” over time (see Clark, 2001).

^3^Readers may be interested to consider the studies that examine the “musical” properties of stone artifacts and acoustical characteristics of Paleolithic environments (see Blake and Cross, 2008).

^4^This involves the integration of phonemes and words into grammatical structures and the development of a generative syntax that provides the “rules” for such processes—or, likewise the organization of discrete sets of sounds, tones, and pitches into rhythmic/formal hierarchies that could be consciously repeated or manipulated (e.g., melodies and drumming patterns).

^5^For studies on music and brain plasticity (see Large and Jones, 1999; Jäncke et al., 2001; Pantev et al., 2001; Schlaug, 2001; Münte et al., 2002; Gaser and Schlaug, 2003; Lappe et al., 2008). Additionally, clinical studies have demonstrated music's deep effects on the body as well as its capacity to transform or reorganize neural structures (e.g., Bunt, 1994; Standley, 1995; Nayak et al., 2000; Tomaino, 2009; Jovanov and Maxfield, 2011).

^6^See Toiviainen (2000) for a discussion of this in the context of music AI.

^7^It should be noted enactivism also has an antecedent in work by Bruner (1964) who coined the term.

^8^The symbiotic and co-emergent relationship between honeybees and flowers is an excellent example of this. Here autonomous organisms exist as environments to each other—the development of their phenotypes are inextricably enmeshed over evolutionary time (Varela et al., 1991; Hutto and Myin, 2012).

^9^This, of course, is not to say that the brain does not play an important role cognitive processes. However, from the enactive perspective, cognition is not limited to the brain—brain, body, and world are different aspects of an integrated cognitive system that functions in a non-linear way. Barrett (2011, p. 57–93) offers many examples that show how creatures with simple neural organizations are nevertheless able to engage in complex intelligent behaviours by using their bodies and environmental features as part of their cognitive systems (see also Brooks, 1991). In line with this, DST research into forms of problem solving and cognitive development associated with coordinated bodily activity have revealed that many of these processes can also be accurately described without necessarily having to recruit representational content (Thelen and Smith, 1994; Kelso, 1995; Chemero, 2009). Indeed, the DST equations employed to model such phenomena are neutral regarding representations. It is argued, therefore, that evoking representation may introduce unnecessary complications (see Chemero, 2009, p. 68–75).

^10^Among other things, this orientation has begun to offer insights into the ways the basic goal directed and self-organizing dynamics discussed above might be extended to living musical situations that are not life threatening in the literal sense, but that nevertheless require constant care and attention to maintain. For example, think of a performing string quartet. Each member must continuously adapt to the evolving musical environment, drawing on different forms of embodied, emotional-affective/cognitive capacities to communicate, develop shared affordances, and maintain the musical ecology they co-create (this example is developed in detail by Salice et al., 2017; see also Krueger, 2014; Schiavio and Høffding, 2015). Similar studies by Walton et al. (2014, 2015) draw on enactive and dynamical systems theory to better understand the real-time dynamics of interacting musical agents in creative improvisational contexts.

^11^A relevant example of approaches involving the integration of video and audio documentation, and DST/4E analysis, may be found in the recent work by Walton et al. (2014, 2015) that examines perceptions of creativity in interacting musical improvisers (see also Borgo, 2005; Laroche and Kaddouch, 2015). Note that these studies also include a phenomenological dimension that incorporates first-person accounts of the participants.
